# Combined Nanofibrous Face Mask: Co-Formulation of Lipases and Antibiotic Agent by Electrospinning Technique

**DOI:** 10.3390/pharmaceutics15041174

**Published:** 2023-04-07

**Authors:** Diána Balogh-Weiser, Alexandra Molnár, Gergő D. Tóth, Gábor Koplányi, József Szemes, Balázs Decsi, Gábor Katona, Maryana Salamah, Ferenc Ender, Anita Kovács, Szilvia Berkó, Mária Budai-Szűcs, György T. Balogh

**Affiliations:** 1 Department of Organic Chemistry and Technology, Budapest University of Technology and Economics, Műegyetem rkp. 3, H-1111 Budapest, Hungary; 2 Department of Physical Chemistry and Materials Science, Budapest University of Technology and Economics, Műegyetem rkp. 3, H-1111 Budapest, Hungary; 3 Institute of Pharmaceutical Technology and Regulatory Affairs, University of Szeged, Eötvös u. 6, H-6720 Szeged, Hungary; 4 Istitute of Pharmacodynamics and Biopharmacy, Faculty of Pharmacy, University of Szeged, Eötvös u. 6, H-6720 Szeged, Hungary; 5 Department of Electron Devices, Budapest University of Technology and Economics, Műegyetem rkp. 3, H-1111 Budapest, Hungary; 6 SpinSplit LLC, Vend u. 17, H-1025 Budapest, Hungary; 7 Department of Chemical and Environmental Process Engineering, Budapest University of Technology and Economics, Műegyetem rkp. 3, H-1111 Budapest, Hungary

**Keywords:** nanoformulation, nano mask, electrospinning, skin treatment, acne vulgaris, lipase

## Abstract

The application of enzyme-based therapies has received significant attention in modern drug development. Lipases are one of the most versatile enzymes that can be used as therapeutic agents in basic skin care and medical treatment related to excessive sebum production, acne, and inflammation. The traditional formulations available for skin treatment, such as creams, ointments or gels, are widely applied; however, their use is not always accompanied by good drug penetration properties, stability, or patient adherence. Nanoformulated drugs offer the possibility of combining enzymatic and small molecule formulations, making them a new and exciting alternative in this field. In this study polymeric nanofibrous matrices made of polyvinylpyrrolidone and polylactic acid were developed, entrapping lipases from *Candida rugosa* and *Rizomucor miehei* and antibiotic compound nadifloxacin. The effect of the type of polymers and lipases were investigated, and the nanofiber formation process was optimized to provide a promising alternative in topical treatment. Our experiments have shown that entrapment by electrospinning induced two orders of magnitude increase in the specific enzyme activity of lipases. Permeability investigations indicated that all lipase-loaded nanofibrous masks were capable of delivering nadifloxacin to the human epidermis, confirming the viability of electrospinning as a formulation method for topical skin medications.

## 1. Introduction

Important life-sustaining processes need enzymes [1] to proceed at a sufficient speed under physiological circumstances, maintaining a low barrier to activation energy. The natural role of enzymes in human skin has always been a widely researched topic. The borderlines between cosmetics and drugs have become increasingly blurred due to the ever more advanced understanding of human physiology. As physiological processes are based on catalytic activity of enzymes, these biocatalysts are also essential for health and body care [2]. Enzymes break up complicated inactive molecules in the skin and convert them into more straightforward and frequently active ones. Proteins can be hydrolyzed or decoupled by proteases, whereas tyrosinase makes melanin formation easier and glycosidases encourage the enrichment of ceramides in the epidermis [3]. Enzymatic reactions operate on the metabolism of sebaceous glands and adipocytes, regulate keratinization processes, ensure inter-corneocytar cohesion, promote tanning and auto-photo protection, act on the whitening activities of age spots, activate the skin’s inherent defense mechanisms, and preserve collagen and elastin fibers on the skin and stratum corneum [4]. The application of enzymes in dermatology and cosmetology has been known for a long time [5,6,7]. However, novel enzyme-based procedures are continuously introduced in modern medicine. Most commonly, enzymes are applied subcutaneously and topically. For example, in dermatologic surgery, the addition of hyaluronidase with regional block anesthetics (procaine, lidocaine, etc.) became popular due to their enhancing effect on the bioavailability of active substances [8]. Studies about the application of photolyase from different organisms showed that this enzyme can remove UVB-induced cyclobutene-pyrimidine dimers, which plays a key role in immunosuppression and photo-carcinogenesis. Photolyase encapsulated in liposomes could be used as an active ingredient in advanced skin care products [9]. Lipases are enzymes that naturally catalyze the cleavage of ester bonds. Their synthetic activity predominates in organic solvents [10] and their hydrolytic activity comes forth in aqueous environments [11]. Prior to encountering the water–oil interface, water-soluble lipases are inactive. Lipases are of great interest in the pharmaceutical [12], food [13], detergent [14], leather [15], and oil industries [16]. The use of lipases is widespread in the pharmaceutical industry, since several chiral intermediates or drug molecules, such as non-steroid anti-inflammatory and antiviral agents, antibiotics, vitamins, anti-tumor, and anti-allergic compounds could be produced by lipase biocatalysts [17,18,19,20,21,22,23,24,25,26,27]. Lipases are commonly used in the enzyme replacement therapy (ERT) of patients with digestive and pancreatic disorders (typical diseases include chronic pancreatitis, pancreatic insufficiency, and pancreatic cancer) [28,29,30]. The aim is to compensate for enzyme deficiencies in the patient’s body with exogenous proteins before health complications arise due to poor digestion. Lipases are primarily used in dermatology and cosmetic applications to help break down fat deposits and to help loosen and remove debris and/or small flakes of dead corneous skin (i.e., peeling), frequently in conjunction with other enzymes such as proteases. Active lipase can be found in products aimed at anti-cellulite treatment or general surficial cleansing [2,31,32]. The proper preparation of the enzyme is a process that is crucial when lipases are used as both active agents and components in cosmetic formulations. Lipases that are intended to be used as active components in cosmetic products are typically encapsulated in micro- or nanoparticles. It has also been observed that storing lipases as dry powder until usage maintains enzyme activity and product composition. Lipase encapsulation particles frequently include agar, as well as a core substance in which the enzymes are distributed, in an oily dissipation medium [33].

The role and functional mechanism of the human skin microbiome is generally poorly understood. However, *acne vulgaris* (acne) is a common skin disorder, which affects approximately 80% of the population of adolescents in varying stages; mechanistic information about this inflammatory skin disease is insufficient [34]. The number of adult patients suffering from acne is also increasing. Acne is a chronic problem, which appears when dead skin cells and oil from the skin clog hair follicles, causing inflammation. Typical symptoms could be blackheads, whiteheads, pimples, sebaceous oily skin, and, sometimes, scarring [35,36]. The face, upper part of the chest, and back can be affected; acne of the face may lead to reduced confidence, anxiety, and, in extreme cases, depression. Acne is caused by several factors, such as acne-inducing bacteria, follicular epidermal hyperproliferation, and excess sebum production. Topical formulas with antibiotics are considered as a first-line treatment against acne. Several types of antibiotics are involved in the form of gels, swabs, and solutions [37]. For acne treatment besides antibiotics, phototherapies can be possible alternatives. The encapsulated active agents in liposomes can be activated by lipase-mediated processes in the epidermis [38,39]. The reduction of lipase activity in acne to moderate excessive sebum production is also targeted by specific inhibitors [40]. However, lipase, as a bioactive agent, can also be found in certain anti-acne formulations. The application of lipases could help to reduce the fatty or oily layer on the skin surface, leading to better skin conditions and bioavailability of potential active substances [33].

Electrostatic fiber formation (electrospinning) is becoming one of the most promising processes for creating nanoscale supports. Its application allows for the creation of solid fibrous polymer structures with exceptionally high specific surfaces with a submicron diameter [41]. These structures could immobilize biological agents to create unique medicine formulations, create filtration-ready synthetic membrane structures, and even create fabrics for apparel [42,43,44]. Electrostatic repulsion takes place between the identically charged polymer particles and elongates the polymer solution droplet when electric potential in the range of kilovolts is applied to the solution emitter. Jet formation suddenly emerges from the surface of the elongated droplet (also known as the Taylor cone) at a critical point, allowing a solid nanoscale fibrous product to be collected on the electrically grounded collector plate. Nanofibers can be utilized for the entrapment as well as the surface immobilization of enzymes. The two main benefits of the former are that the immobilization procedure may be completed in a remarkably short amount of time and that the polymer matrix created during immobilization surrounds the trapped proteins, offering high levels of environmental protection [45,46].

**Figure 1:**
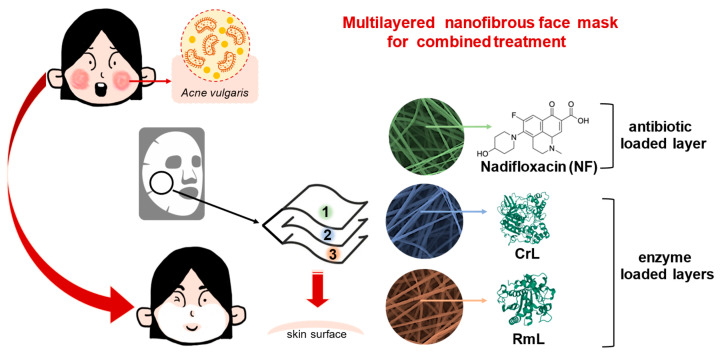
The composition of a multilayered nanofibrous face mask for combined treatment of *acne vulgaris*. The three-layered mask is built up of two enzyme loaded layers: lipase from *Candida rugosa* (CrL) and lipase from *Rhizomucor miehei* (RmL) formulated in polymeric (PVP or PLA) nanofibers and one layer with antibacterial effect: nadifloxacin (NF) formulated in polymeric nanofibers.

The aim of this research Is to introduce a prototype of a combined nanostructured face mask produced by electrospinning technique. In this study, a water-soluble matrix from polyvinylpyrrolidone (PVP) and a water-resistant matrix from polylactic acid (PLA) were formed containing an immobilized mixture of different lipases (CrL: lipase from *Candida rugosa* and RmL: lipase from *Rizomucor miehei*) and an antibiotic agent, nadifloxacin (NF) (Figure 1). Nadifloxacin, primarily as topical fluoroquinolone, is effective in treating Gram-negative, Gram-positive, and anaerobic bacteria superficial skin infections [47]. When applied against *acne vulgaris* as a potential therapeutic target, the role of the lipase is to aid the penetration of the active ingredient by mitigating the patient’s increased sebum production. In this study, the lipase is carried by the nanofibrous mask applied directly on the face skin of the patient. The effect of the nanofibrous formulation on the entrapped lipases were investigated in the lipase catalyzed hydrolysis of three natural fatty acid methyl esters (palmitic acid, linoleic acid, and oleic acid), which are dominant components of the human skin lipid composition [48]. The morphological and structural homogeneities were investigated by scanning electron and Raman microscope, wetting behavior and elasticity of nanofibers were studied by contact angle measurement and mechanical stress investigations. The penetration of the antibiotic agent was examined with a vertical Franz diffusion cell system involving heat-separated epidermis from human abdominal skin.

## 2. Materials and Methods

### 2.1. Materials

Polylactic acid (PLA, Mw = 68 kDa), polyvinylpyrrolidone (PVP, Mw = 360 kDa), lipase from *Candida rugosa* (CrL), lipase from *Rhizomucor miehei* (RmL), palmitic acid (**PA**), oleic acid (**OA**), linoleic acid (**LA**), hydrochloride acid (HCl 37%, *w*/*w*), methanol, toluene, hexane, ethanol, sodium phosphate monobasic, deuterated chloroform (CDCl_3_), dichloromethane (DCM), dimethylformamide (DMF), methyl heptadecanoate were purchased from Merck (Darmstadt, Germany). Nadifloxacin was obtained from TCI (Rockenberg, Germany). In all cases, the water was purified by a Millipore Milli Q water purification system (Bradford, MA, USA). 

### 2.2. Production of Fatty Acid Methyl Esters (FAMEs) for Lipase Activity Analysis

Then, 5.0 g of the appropriate fatty acid (**PA**, palmitic acid; **LA**, linoleic acid; or **OA**, oleic acid) was placed in a 250 mL round-bottom glass flask and dissolved in 10.0 mL of toluene. To the obtained solution, 75.0 mL of methanol and 15.0 mL of the HCl stock solution (9.7 mL of HCl was diluted with 41.5 mL of methanol) were added in this order. The reaction mixture was stirred and refluxed at 100 °C for 1.5 h by a magnetic stirrer (IKA RH basic, IKA GmbH, Staufen im Breisgau, Germany). After cooling to room temperature, for extraction of the FAME 50 mL of hexane and 50 mL of water were added to the reaction mixture. The organic phase with FAME (**PA-ME**, **LA-ME,** or **OA-ME**) was then evaporated at 60 °C and 40 Torr using a rotary vacuum separator (Rotavapor R-114 paired with Waterbath B-480, Büchi, Flawil, Switzerland). The structural analysis and purity of the product was investigated by NMR analysis.

### 2.3. NMR Analysis of Fatty Acid Methyl Esters (FAMEs)

Then, 30 mg of the appropriate fatty acid methyl ester (**PA-ME**, **LA-ME,** and **OA-ME**) was dissolved in 1 mL of CDCl_3_ by vigorous shaking for 1 min by vortex (IKA Mixer Vortex Shaker MS 2, IKA GmbH, Staufen im Breisgau, Germany). The obtained solution was introduced into an NMR tube and proton NMR spectra was measured by a 500 MHz multinuclear NMR spectrometer (Bruker Avance, Billerica, MA, USA). Signals are given in ppm on the δ scale for FAMEs. ^1^H-NMR (CDCl_3_) for **PA-ME**: δ 3.69 (s, 3H), 2.32 (t, *J =* 7.6 Hz, 2H), 1.64 (p, *J =* 7.2 Hz, 2H), 1.36–1.24 (m, 24H), 0.90 (t, *J =* 6.9 Hz, 3H); for **LA-ME**: δ 5.44–5.31 (m, 4H), 3.69 (s, 3H), 2.79 (t, *J =* 6.7 Hz, 2H), 2.32 (t, *J =* 7.6 Hz, 2H), 2.06 (q, *J =* 6,9 Hz, 4H), 1.43–1.26 (m, 16H), 0.91 (t, *J =* 6.8 Hz, 3H); and for **OA-ME**: δ 5.43–5.33 (m, 2H), 3.69 (s, 3H), 2.33 (t, *J =* 7.6 Hz, 2H), 2.08–2.00 (m, 4H), 1.72–1.57 (m, 2H), 1.41–1.27 (m, 20H), 0.91 (t, *J =* 6.9 Hz, 3H). The NMR spectra obtained from the characterization of the fatty acid methyl esters showed a high degree of correspondence with the spectra reported in the H1 NMR library [49,50,51].

### 2.4. Preparation of Nanofibrous Masks by Electrospinning Technique

#### 2.4.1. Preparation of Lipase-Containing PLA and PVP Precursors for Electrospinning

For entrapment of lipases in PLA and PVP nanofibers, an 8.87 wt% PLA solution in the mixture of DCM and DMF (6:1, *v*/*v*) (*solution A*), a 26.47 wt% PVP solution in MilliQ water (*solution B*) and a 9.09 wt% lipase (CrL or RmL) solution in MilliQ water (*solution C*) were prepared. Precursor solutions for electrospinning were prepared by mixing 902 mg of *solution A* with 98 mg of *solution C* for PLA and by mixing 755 mg of *soluiton B* with 245 mg of *solution C* for PVP. Precursors thus obtained were homogenized by vigorous shaking for 3 min by vortex (IKA Mixer Vortex Shaker MS 2, IKA GmbH, Staufen im Breisgau, Germany). Polymer contents of the obtained precursors were 8 wt% and 20 wt% for PLA and PVP, respectively. In all precursors, lipase accounted for 10 wt% of solid matter content (*m*_lipase_ × (*m*_polymer_ + *m*_lipase_)^−1^). Formulation by electrospinning was carried out according to Section 2.4.3.

#### 2.4.2. Preparation of Nadifloxacin-Containing PLA and PVP Precursors for Electrospinning

In order to formulate nadifloxacin into PVP nanofibers, 31 mg of solid nadifloxacin was added to 5 g of 20 wt% aqueous PVP solution. For formulating nadifloxacin into PLA nanofibers, 5 mg of solid nadifloxacin was added to 2 g of 8 wt% PLA solution (in DCM:DMF (6:1, *v*:*v*)). In all precursors, nadifoxacin accounted for 3 wt% of solid matter content (*m*_nadifloxacin_ × (*m*_polymer_ + *m*_nadifloxacin_)^−1^). The obtained precursors were homogenized by vigorous shaking for 5 minutes by vortex. After homogenization, the formulation by electrospinning was carried out according to Section 2.4.3.

#### 2.4.3. Formulation of Nadifoxacin and Lipases into PLA and PVP Nanofibers by Electrospinning

Homogenized precursor mixtures (see Section 2.4.1 and Section 2.4.2) were introduced airtight into a sterile syringe (1 mL Injekt-F Solo, B Braun, Melsungen, Germany), and the electrospinning process was carried out with the Spincube (Spinsplit LLC, Budapest, Hungary) laboratory electrospinning device using a single needle emitter. During the electrospinning experiments 20 kV of voltage was applied to the precursors at 15 cm collector-emitter distance and 20 μL min^−1^ feed rate using HydroDrive to minimize precursor waste. The experiments were carried out at room temperature. Nanofibrous samples were stored at 4 °C in a refrigerator prior to use. The electrospinning process resulted in a lipase content (loading) of 10 wt% in all the nanofibrous products.

#### 2.4.4. Preparation of Multi-Layered Nanofibrous Face Masks by Electrospinning

A three-layer mask was created with a bottom layer containing 10 wt% CrL, a middle layer containing 10 wt% RmL and a top layer containing 3 wt% nadifloxacin (corresponding to 1 wt% by weight of the final product). The mass of each layer of the masks was 120 mg, which was regularly checked during production. Enzyme loaded layers were produced based upon precursors described in Section 2.4.1 and the nadifloxacin loaded layer was performed based upon precursors prepared according to Section 2.4.2. Once the desired layer mass was achieved, the new layer was electrospun directly on top of the previous one, with the process parameters detailed in Section 2.4.3.

### 2.5. Morphological Characterization by SEM

The morphology of nanofibers was studied by a JSM JEOL-5500LV (JEOL, Tokyo, Japan) SEM-EDS scanning electron microscope (SEM). Samples were placed on a copper grid and to ensure adequate surface conductivity of the samples, they were coated with gold in a few atomic layers of thickness by a nebulizer (Polaron sc760 mini sputter coater, Thermo VG Microtech, Waltham, MA, USA) using Argon-plasma, at 10 mA for 180 s. The images were taken in the high vacuum mode. The acquired images were processed using digital image evaluation software (ImageJ 1.52r, U.S. National Institutes of Health) to determine the average diameter and standard deviation of the nanofibers (*n* = 100 measurement points).

### 2.6. Structural Characterization by Raman Microscopy

Raman mapping of nanofibers was performed using a Thermo Fisher DXR dispersive Raman instrument (Thermo Fisher Scientific Inc., Waltham, MA, USA) equipped with a CCD camera and a diode laser operating at a wavelength of 780 nm. For sample preparation, a glass slide was covered with aluminum foil then mounted with electrospun nanofibers. A Raman chemical map was obtained from a 100 × 100 µm surface of different nanofibers with a 1×1 µm spectral resolution while applying a laser power of 12 mW at a 50 µm slit aperture size. The spectrum of the chemical map was recorded with an exposure time of 2 s and acquisition time of 6 s, for a total of 32 scans per spectrum in the spectral range 3300–200 cm^−1^ with cosmic ray and fluorescence corrections. Each Raman map was normalized in order to eliminate the intensity deviation between the measured areas. The distribution of enzymes and nadifloxacin was investigated with ImageJ 1.4 software (National Institutes of Health, Bethesda, MD, USA) by measuring the net area of Raman maps, where the Raman spectra characteristic for enzymes and nadifloxacin are located.

### 2.7. Determination of the Elastic Modulus of Nanofibers

Single direction stress–strain measurements were performed on an Instron 5543 (Norwood, MA, USA) mechanical tester equipped with a 10 N load cell at room temperature. To secure the nanofibrous samples, 4 × 4 cm squares of printer paper were cut with a 2 × 2 cm window in the middle. The nanofibrous samples (cut into 1 × 3 cm pieces) were placed between two paper frames, then the resulting assembly was glued together with superglue (Loctite, Düsseldorf, Germany) and left to dry overnight. The prepared samples were secured in the measuring apparatus with a 2 cm spacing and the sides of the frame were cut with scissors before the measurement was started. For the modulus tests, the samples were pulled at a speed of 10 mm min^−1^. All measurements were performed in triplicate. 

### 2.8. Water Contact Angle Measurement

The water contact angle of fiber mats was determined by a circle fitting method using OCA Contact Angle System (Dataphysics OCA 20, Dataphysics Inc., GmbH, Germany). The liquid medium used for our contact angle measurements was bidistilled water (interfacial tension (γp = 72.8 mN/m). The surface-free energy of nanofibers were determined by Wu method (harmonic mean). The samples were measured in triplicate, data were presented as means ± SD.

### 2.9. Measurement of Enzymatic Activity

In a 4.0 mL glass vial, 10.0 mg of native lipase, or 5.0 mg of nanofibrous lipase formula (lipase entrapped in PLA or PVP nanofibers, or lipase containing three-layer mask), was measured, then 1.0 mL buffer solution (sodium phosphate, 50 mM, pH 7.5) was added and the mixture was shaken for 10 min at room temperature. Afterwards, 200 μL of a substrate containing the reaction mixture (50 μL from the proper fatty acid methyl ester, **PA-ME**, **LA-ME,** or **OA-ME** in 150 μL ethanol) was added to the mixture, then it was shaken at 33.0 °C (average temperature of human skin surface) on an orbital shaker (450 rpm; Heidolph TitraMax 1000, Schwabach, Germany). After 2, 5, and 24 h, the 25 μL sample was taken from each reaction and diluted with 500 μL ethanol. Samples were analyzed according to FAME standard protocols described in the previous study [52]. To each sample, the methyl heptadecanoate (**HD-ME**) solution was added as an internal standard, which resulted in a 0.5 mg mL^−1^ concentration of the standard in all samples. Reaction samples were analyzed by a gas chromatograph (Agilent 5890D with HP-5 (5 m × 0.31 mm × 0.1mm film, 5% phenylmethylpolysiloxane) stationary phase, Santa Clara, CA, USA) applying the following method: 160 °C 5 min; 160–280 °C, 10 °C min^−1^; 280 °C 5 min. The components were identified according to their retention time; *t*_R_ = 8.35 min for **PA-ME**, *t*_R_ = 10.45 min for **LA-ME**, *t*_R_ = 10.45 min for **OA-ME**, and *t*_R_ = 9.70 min for **HD-ME_._** All tests were performed in triplicate. Based on the chromatograms the following parameters were determined, conversion [(*c*; %) *c* =(1 − *M_S_* × *M_S_*_,0_^−1^) × 100], specific biocatalytic activity [(*U_B_*; U × g^−1^) *U_B_* = *n_s_* × *c* × (*t* × *m_B_*)^−1^], specific enzyme activity [(*U_E_*, U × g^−1^) *U_E_* = *n_s_* × *c* × (*t* × *m_E_*)^−1^], where *M_S_* and *M_S_*_,0_ are the actual and the initial (*t* = 0 s) molar amounts of the substrate (S), *n_S_* is the amount of substrate [µmol], *t* is the time elapsed [min], *m_B_* is the mass of the biocatalyst [g], and *m_E_* is the mass of the enzyme [g].

### 2.10. Ex Vivo Skin Permeability Studies

The ex vivo permeability studies were performed with a vertical Franz diffusion cell system (Hanson Microette TM Topical & Transdermal Diffusion Cell System, Hanson Research Corporation, Chatsworth, CA, USA) using a heat-separated human epidermis (HSE). The human skin samples originated from patients who had undergone abdominal plastic surgery. The subcutaneous fatty tissue was removed immediately after excision, and the skin was stored frozen at −20 °C until the application. Before the permeation study, the skin was thawed at room temperature. During the heat separation, the thawed skin samples were immersed in water at 60 °C for 90 s, which enabled the removal of the epidermis from the underlying dermis using forceps. The epidermal membrane was kept in PBS (pH = 7.4) for at least 20 min. After this hydration process, the heat-separated epidermis (HSE) was placed on a supporting Whatman filter paper (Grade 1, size: 2.5 cm, GE Healthcare Life Sciences, UK). The measurements were carried out with the approval of the Hungarian Medical Research Council (ETT-TUKEB, registration number: BMEÜ/2339-3/2022/EKU). To guarantee appropriate quantity of the masks for the ex vivo tests, electrospun mats were folded to a 5-layer sheet, then placed as a donor phase over human epidermis supported on Whatman paper, where the effective diffusion surface area was 1.77 cm^2^. The individual sample amounts were measured. The acceptor phase was 7.0 mL PBS (pH 7.4). Experiments were performed at 32 °C for 24 h. Then, a 0.8 mL sample was taken from the acceptor phase after 0.5, 1, 2, 3, 6, 12, 18, and 24 h, and replaced with fresh PBS. After the 24 h permeation study, the donor phase, the HSE, and the Whatman paper were removed from each Franz cell and HSE and filter paper were extracted with 5 mL methanol:water (50:50) solution. The extracted samples were filtrated using PTFE syringe filter (0.45 µm). Three parallel measurements were carried out; the amount of nadifloxacin was analyzed by means of HPLC.

### 2.11. HPLC Analysis

Quantification of NF concentration in the experiments was performed with an Agilent 1260 (Agilent Technologies, Santa Clara, CA, USA) HPLC. As stationary phase a 250 × 4.6 mm Zorbax^®^ with 5µm particle size (Agilent Technologies, Santa Clara, USA) was applied. The mobile phase consisted of methanol:acetonitrile:water 55:30:15 *v*/*v* adjusted to pH 4 ± 0.5 with orto-phosophoric acid (85% *v*/*v*). The injection volume was 10 µL. The isocratic elution was performed for 5 min at a flow rate of 1.0 mL/min at 40 °C. Chromatograms were detected at 290 nm using UV-VIS diode array detector at the retention time of 3.09 min. The linear regression of the calibration was 0.9999. The limit of quantification (LOQ) and detection (LOD) of NF were 10.628 ppm and 3.507 ppm, respectively. Data were evaluated using ChemStation B.04.03. Software (Agilent Technologies, Santa Clara, USA).

## 3. Results and Discussion

### 3.1. Formulation of Lipases and Nadifloxacin into Polymeric Nanofibers

**Figure 2:**
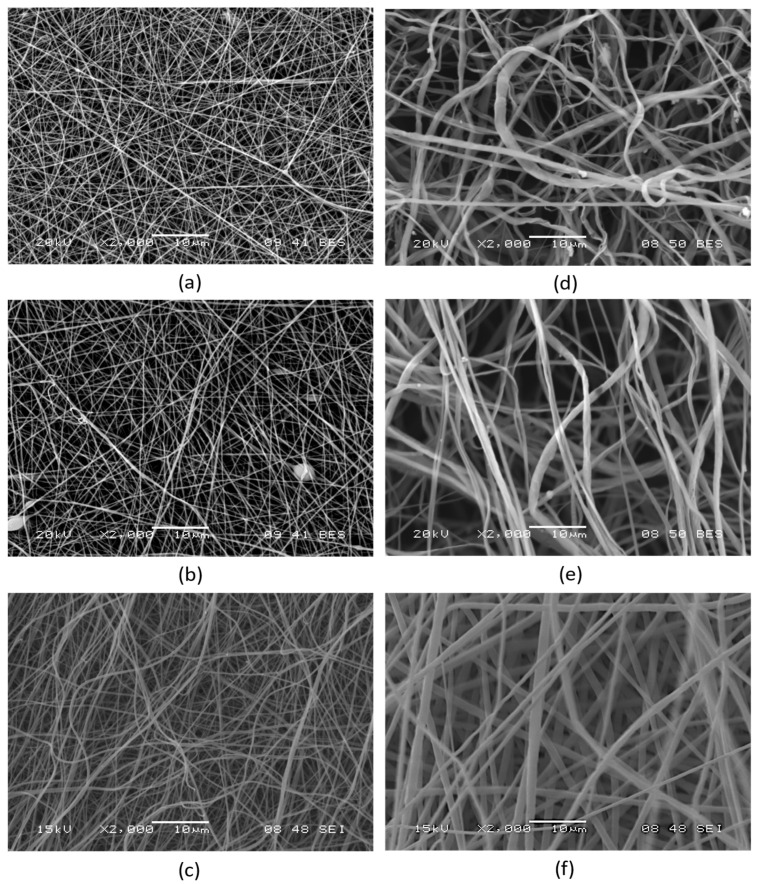
Scanning electron microscope (SEM) images, depicting the morphologies of nanofibrous PVP matrices containing (**a**) 10 wt% CrL; (**b**) 10 wt% RmL; and (**c**) a multi-layered PVP mask consisting of a CrL containing (10 wt%) bottom layer, a RmL containing (10 wt%) middle layer, and a nadifloxacin containing (3 wt%) top layer; as well as nanofibrous PLA matrices containing (**d**) 10 wt% CrL; (**e**) 10 wt% RmL; and (**f**) a multi-layered PLA mask consisting of a CrL containing (10 wt%) bottom layer, a RmL containing (10 wt%) middle layer and a nadifloxacin-containing (3 wt%) top layer and middle layer.

The first step was to evaluate the impact of enzymes (CrL and RmL) and nadifloxacin entrapped in PVP and PLA nanofibers by electrospinning on the fiber morphology. To this end, the electrospinning experiments described in Section 2.4. were carried out, and the fibrous products produced were studied using scanning electron microscopy (SEM), as described in Section 2.5. Table 1 displays the average fiber diameters and standard deviations for the nanofiber samples studied, while Figure 2 depicts SEM images of the same samples. The examination of the electron microscopy results reveals that the structure of the electrospun products is fibrous as expected, with only minor scattered structural flaws. The average diameter of PLA fibers is noticeably larger than that of PVP fibers, and the standard deviation in diameter is likewise greater, but their morphology is nevertheless acceptable (Table 1). In the case of PVP, the entrapped enzymes slightly reduced the average fiber diameter of initial fibers, however nadifloxacin increased it. The fiber diameter of PLA nanofibers was decreased due to the presence of enzymes or antibiotics. Regarding the multilayered mask, PVP- and PLA-based mats had thinner fibers than the “empty” polymeric fibrous matrices. 

Morphological investigations applying SEM showed that all electrospun samples had a fibrous structure with a few heterogenities (Figure 2). We can conclude from studying the structure of the nanofibers that the precursors used are suitable for the creation of the products described in the aim of the study. This is further supported by the fact that, due to the favorable properties of the precursors, the fiber formation was stable at constant parameters during the electrospinning experiments.

### 3.2. Structural Analysis by Raman Microscopy

**Figure 3:**
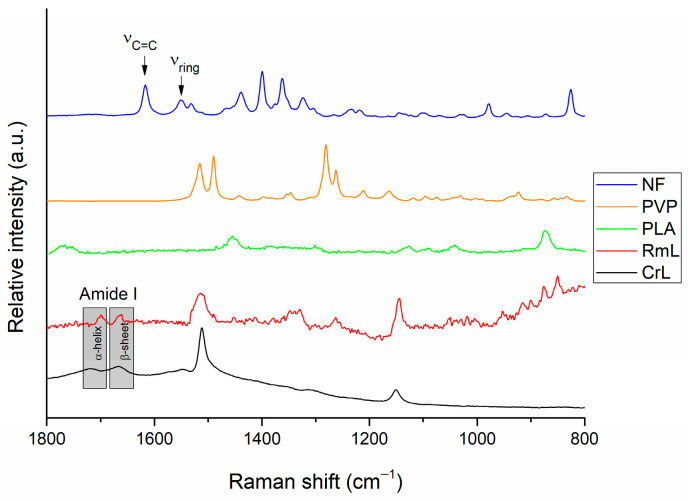
Raman spectra of initial components, NF, nadifloxacin; PVP, polyvinyl pyrrolidone; PLA, polylactic acid; RmL, lipase from *Rizomucor miehei*; and CrL, lipase from *Candida rugosa*.

After the structural analysis, Raman mapping (see Section 2.6) was used to examine the distribution of the formulated agents (lipases and nadifloxacin) across the various nanofibers. In the case of each fiber, mat three randomly selected an area of 100 × 100 µm surface to investigate in order to determine the distribution of nadifloxacin and different lipases (CrL and RmL) (Figure 3). The chemical structure of initial components was investigated with the aim to find unique spectral sections in the Raman spectra characteristic for lipase and nadifloxacin, which can be used as a reference for the localization of the formulated agents. The Raman spectra of non-entrapped lipase and nadifloxacin were used as a reference for the localization of the formulated agents, and their frequency of occurrence is shown in the chemical maps, which represent the statistical distribution of the specific chemical entities. For nadifloxacin profiling, the Raman peak at 1550 cm^−1^ is assigned to the stretching vibration of the quinolone ring system and the Raman band at 1617 cm^−1^ is assigned to the C=C asymmetrical stretching vibration of the aromatic rings as unique spectral vibrations in comparison to other components. In the case of fiber mats containing combined enzyme composition, the differentiation of embedded enzymes was challenging due to the masking of the polymer matrix. Therefore, the profiling was conducted based on the main protein structural element (amide I), which did not overlap with any other spectral vibration of other components. The vibrational bands of carbonyl stretching of amide I region (1600–1700 cm^−1^) shows strong Raman intensity, which corresponds to the secondary structure of lipase. In the Raman spectrum (Figure 3), the characteristic amide I band arises principally from the C–O stretching vibration of the peptide group of lipases. The α-helix (1667 cm^−1^) and β-sheet (1719 cm^−1^) can be clearly observed for both enzymes applied [53]. In addition to amide I region, the C–H bending in the protein structure (1512 cm^−1^) and the amide III region (1147 cm^−1^) of enzymes can be also observed corresponding to N–H bending, C–N stretching of peptide bonds, however similar spectral vibrations were found in case of PLA and PVP, therefore they were not suitable for further analysis.

**Figure 4:**
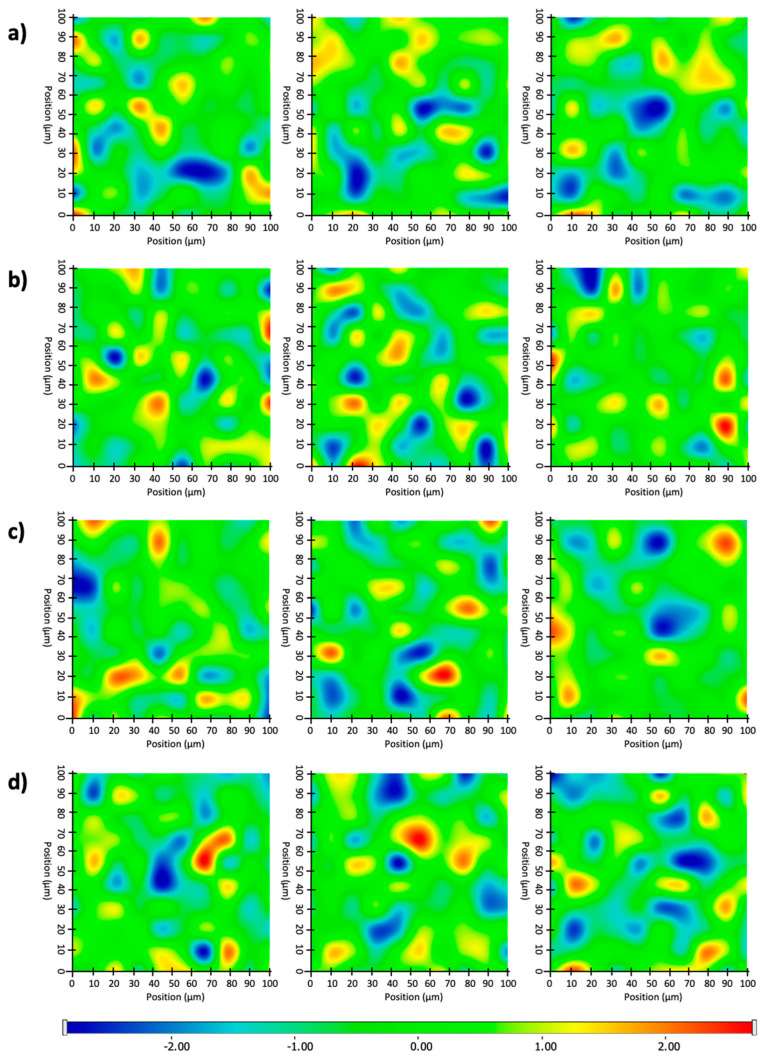
Raman chemical maps of randomly selected areas from three different places of various PVP- and PLA-based electrospun non-woven tissues indicating the distribution and relative occurrence of lipases and nadifloxacin entrapped within them: (**a**) RmL and CrL distribution in PVP nanofiber-based multilayered mask, (**b**) nadifloxacin distribution in PVP nanofiber-based multilayered mask, (**c**) RmL and CrL distribution in PLA nanofiber-based multilayered mask, and (**d**) nadifloxacin (NF) distribution in PLA nanofiber-based multilayered mask.

After the structural examination of initial components, Raman mapping was conducted in order to investigate the distribution of nadifloxacin and different lipases in the fiber mats. For localization, the previously mentioned spectral regions were used as a reference whose frequency of occurrence is shown in the chemical maps, which represent the statistical distribution of the specific chemical entities (Figure 4). The different colors of the chemical map indicate the relative intensity change of the investigated components in the nanofibers. Red color indicates its strong existence, the green area shows a mixed composition, whereas blue color marks those regions of the map whose spectral resolution contains different spectra characteristic of another component. The results revealed that the distribution of nadifloxacin, as well as different lipases, was homogenous in cases of both polymers, as shown by well-defined packages with yellow-red color.

**Figure 5:**
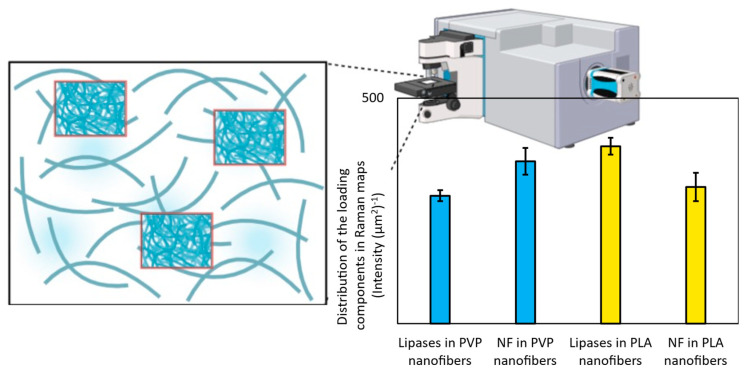
Distribution of lipases (RmL and CrL) and nadifloxacin (NF) in PVP and PLA nanofibers indicating the strong existence of enzymes or antibiotic agent based on their specific Raman intensity.

The distribution of different chemical entities (belonging to lipases and nadifloxacin) was also investigated by the scanning of three different regions of the total sample. The total area of red pixels in each Raman map (where the strong existence of active compounds was detected) was measured by image calculator and the average of three Raman maps recorded from the same fiber mat was plotted (Figure 5). The relatively low standard deviation of randomly recorded Raman maps revealed that both the lipases and nandifloxacin were homogenously distributed in the investigated nanofibers.

### 3.3. Characterization of Nanofibers by Water Contact Angle Measurement and Mechanical Stressing

For a comprehensive investigation of nanofibrous matrices, elastic properties by mechanical stressing and wetting behavior by water contact angle measurements were studied (Table 2). 

To investigate the mechanical properties of the electrospun masks, and to study the effect of the entrapped components on the elasticity of the masks, we compared pure PLA and PVP nanofibers to lipase and antibiotic-containing PLA and PVP multilayer masks in the modulus study described in Section 2.7. The results show that the elastic modulus of PVP nanofibers is higher than that of PLA nanofibers for both pure fibers and multi-layered masks. Furthermore, the entrapped components had a softening effect on the nanofibers. This observation supports the previously presented theory that interaction occurs between the polymer matrices of the nanofibers and the entrapped components.

The water contact angle (CA) measurement (see Section 2.8) revealed that PVP-based nanofibers have reduced hydrophobicity (CA_water_ = 5.35 ± 0.9°), in comparison to PLA fiber mats; moreover, due to the high specific surface area, fast dissolution is expected. The enzyme and nadifloxacin embedded PVP nanofibers showed slightly higher hydrophobicity (CA_water_ = 11.69 ± 1.2°), which can be claimed with the lipophilic nature of nadifloxacin, however this hydrophobicity value still predicts rapid dissolution. In cases of PLA nanofibers, the CA was higher than 90°, which indicates high hydrophobicity, and therefore sustained drug release. Nadifloxacin and enzymes slightly decreased the CA, but the hydrophobic properties of PLA were still preserved. The measured surface free energy values support the same findings, they are inverse proportional with the CA values. High surface free energy value indicates improved wetting properties.

### 3.4. Effect of Nanofibrous Formulation of Enzymatic Activity

**Figure 6:**
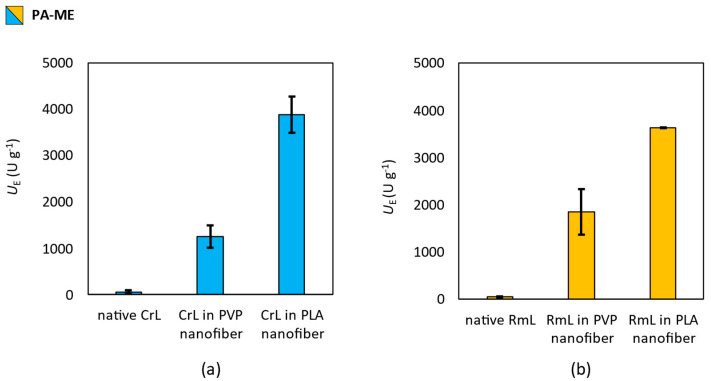
Investigation of the effect of entrapment by electrospinning into PVP and PLA nanofibers on the biocatalytic activity of (**a**) lipase from *Candida rugosa* (CrL) and (**b**) lipase from *Rhizomucor miehei* (RmL) in the biocatalyzed hydrolysis of methyl palmitate (**PA-ME**). Test reactions were carried out at 33 °C constant shaking. This figure summarizes the specific enzyme activity values obtained by gas chromatography of samples taken 2 h after the start of the reactions. Activity values were determined by the following equation: *U*_E_ = *n*_s_ × *c* × (*t* × *m*_E_)^−1^, where *c* is the conversion (%) *n*_S_ is the amount of substrate [µmol], *t* is the time [min] and *m*_E_ is the mass of enzyme [g].

**Figure 7:**
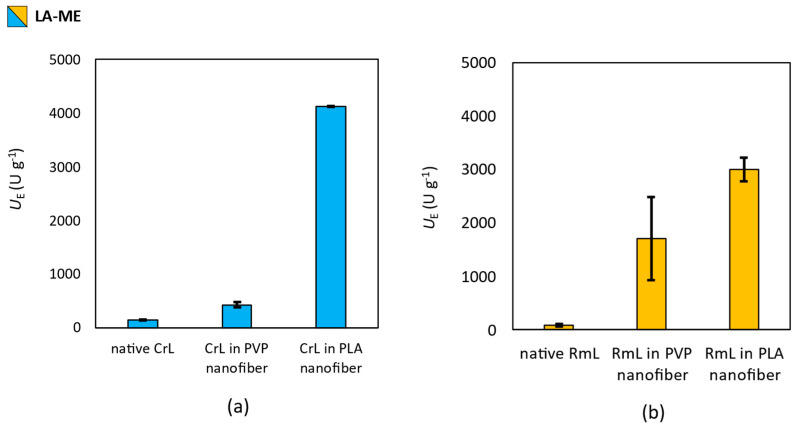
Investigation of the effect of entrapment by electrospinning into PVP and PLA nanofibers on the biocatalytic activity of (**a**) *Candida rugosa* (CrL) and (**b**) *Rhizomucor miehei* (RmL) lipases in the biocatalyzed hydrolysis of methyl linoleate (**LA-ME**). Test reactions were carried out at 33 °C under constant shaking. This figure summarizes the specific enzyme activity values obtained by gas chromatography of samples taken 2 h after the start of the reactions. Activity values were determined by the following equation: *U*_E_ = *n*_s_ × *c* × (*t* × *m*_E_)^−1^, where *c* is the conversion (%) *n*_S_ is the amount of substrate [µmol], *t* is the time [min], and *m*_E_ is the mass of enzyme [g].

**Figure 8:**
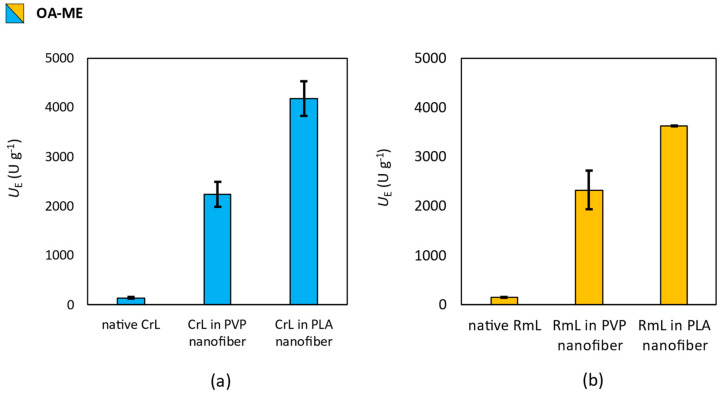
Investigation of the effect of entrapment by electrospinning into PVP and PLA nanofibers on the biocatalytic activity of (**a**) *Candida rugosa* (CrL) and (**b**) *Rhizomucor miehei* (RmL) lipases in the bi-catalyzed hydrolysis of methyl oleate (**OA-ME**). Test reactions were carried out at 33 °C under constant shaking. This figure summarizes the specific enzyme activity values obtained by gas chromatography of samples taken 2 h after the start of the reactions. Activity values were determined by the following equation: *U*_E_ = *n*_s_ × *c* × (*t* × *m*_E_)^−1^, where *c* is the conversion (%) *n*_S_ is the amount of substrate [µmol], *t* is the time [min], and *m*_E_ is the mass of enzyme [g].

To investigate the effect of the electrospinning technique and the polymer (PVP or PLA) used as a matrix on the activity of the lipases (CrL or RmL) entrapped in the nanofibers, the activity of immobilized enzymes was tested in the hydrolysis of fatty acid methyl esters (see in Section 2.9). Lipases were able to hydrolyze the methyl esters of the most common saturated (palmitic acid), mono-unsaturated (oleic acid) and di-unsaturated (linoleic acid) fatty acids found on the human skin surface was tested. The results of the activity test are summarized in Figure 6, Figure 7 and Figure 8.

Results with the hydrolysis of palmitic acid methyl ester (**PA-ME**) showed that CrL and RmL entrapped in polymeric nanofibers had larger specific enzyme activity (*U*_E_) than their non-immobilized, native form (Figure 6). PLA-based nanofibers enhanced the specific enzymatic activity (*U*_E_) more than PVP for both types of lipases.

In the case of linoleic-acid ester (**LA-ME**), similar observations could be described, but the difference between PVP and PLA for CrL entrapment is larger than that was given in PA-ME hydrolysis (Figure 7a). Although, PVP or PLA for RmL immobilized had moderate effect on *U*_E_ (Figure 7b).

Regarding the lipase catalyzed hydrolysis of oleic acid methyl ester (**OA-ME**), results were similar to **PA-ME**, the remarkably positive effect of nanofibrous formulation was clearly seen on *U*_E_ values. The difference between PVP and PLA matrices and the two investigated lipases (CrL and RmL) was moderate (Figure 8a,b).

Based on the results presented in Figure 6, Figure 7 and Figure 8, the following conclusions could be presented. Native CrL showed higher activity than RmL for two substrates. A 10% higher conversion was achieved in the hydrolysis of PA-ME and 30% higher in the hydrolysis of LA-ME using CrL. In the case of OA-ME, the catalytic activity of the two lipases was identical. 

Electrospinning resulted in a clearly observable improvement in the catalytic activity of both lipases, regardless of the polymer used as the matrix. In comparison to the native case, RmL has shown greater activity in the hydrolysis of PA-ME and LA-ME when using the PVP matrix, but in the hydrolysis of OA-ME, the two enzymes still showed equal activity. When PLA matrix was used, the activity of CrL was higher in all the examined cases. The change in activities can be explained by the development of energetically favorable interactions between the matrix and the protein. This explanation is supported by the fact that the use of polymer matrices of different quality had different effects on the activity of the two different lipases. To prove the existence of these interactions, and to characterize their properties, molecular dynamics calculations would be necessary, which is beyond the scope of this publication.

In the morphological studies, we could see that the diameter of PVP nanofibers is visibly smaller than that of PLA nanofibers. One would expect from the larger specific surface area of PVP nanofibers that the catalytic activity of the lipases entrapped into them would be higher, but the situation is not so simple. In addition to the specific surface area of the nanofibers, the activity of the immobilized lipases is largely determined by the strength of the stabilizing interactions between the polymer matrix and the lipase, as well as the water solubility of the formulation. Our studies have shown that the use of the PLA matrix was always associated with a substantially larger increase in enzyme activity than the use of the PVP matrix. This is most likely explained by the fact that the PVP matrix dissolves in the aqueous reaction medium as opposed to the PLA matrix. The consequence of the dissolution of the PVP matrix is that the favorable microenvironment formed around the protein in the nanofiber loosens, decomposes and is, thus, no longer able to stabilize lipase to such a high degree. In the case of the PLA matrix, the water insolubility likely contributes to the perseverance of the microenvironment that stabilizes the lipase, therefore these preparations are capable of exerting higher activity. The diversity in the strength of polymer–lipase interactions may also be involved in the different enzyme activities that can be achieved using the various polymer matrices, but on this topic no conclusive statement can be made due to the different water solubility of the polymers. The state of the catalyst engaged in the reaction (homogeneous or heterogeneous catalysis) has a considerably greater influence on the observed activity than the strength of the lipase–polymer interaction.

To investigate the effect of the presence of nadifloxacin on the activity of lipases immobilized in polymeric nanofibers, the activity assay described in Section 2.9 was also performed with the three-layer nanofibrous face masks (see Section 2.4.4). Compared to the single-layer lipase containing nanofibers discussed earlier, the lipase:polymer mass ratio in face masks was modified in favor of the polymer due to the presence of the upper nadifloxacin-containing layer. For this reason, a three-layer face mask was also produced of both matrices with no nadifloxacin in the top layer (consisting of only the pure polymer nanofibers) and the activity of this formula was also investigated in order to more accurately explore the effect of the nadifloxacin loading. A comparison of the biocatalytic activities of different single-layer and three-layer lipase containing nanofiber formulations is presented in Table 3.

Table 3 shows that the biocatalytic activity (*U*_B_) of three-layer preparations was typically lower than that of single-layer preparations. This can be explained by the presence of the top lipase-free layer in the three-layer masks. Although the lower two layers both contain 10 wt% lipase, due to the presence of the upper lipase-free layer, the preparation as a whole has an enzyme content of only 6.67 wt%, compared to 10 wt% for single-layer preparations. 

The other observation is that the presence of nadifloxacin in three-layer masks has no effect on the biocatalytic activity of the entrapped lipases during the hydrolysis of **LA-ME** and **OA-ME**, however, it has caused a deterioration in enzyme activity in the hydrolysis of **PA-ME**. The activity-deteriorating effect of nadifloxacin is presumably caused by an unfavorable lipase–active substance interaction, but its characterization is beyond the scope of this publication. Identifying this interaction also requires the use of computational chemistry. It is also important to notice that this effect was visible in both of the different quality polymer matrices, but to a considerably lesser extent in the PLA matrix. This phenomenon is likely also related to the matrix’s water insolubility, as the matrix persisting in the reaction media serves to separate lipases in space from the activity reducing nadifloxacin.

### 3.5. Investigation of Drug Penetration in Human Skin

**Figure 9:**
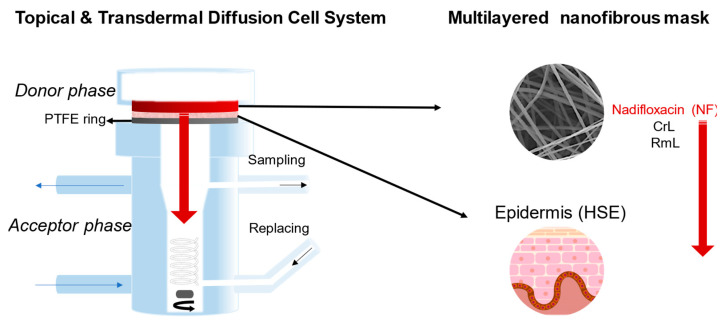
Experimental set up of skin permeability test of nanofibrous mask (multilayered mask containing nadifloxacin, CrL and RmL enzymes) using a topical and transdermal diffusion cell.

For the investigation of nadifloxacin penetration on human skin, a Franz diffusion cell was applied, in which a heat-separated epidermis (HSE) was placed (see details in Section 2.10). Nanofibrous samples were fitted on to the HSE layer (Figure 9).

**Figure 10:**
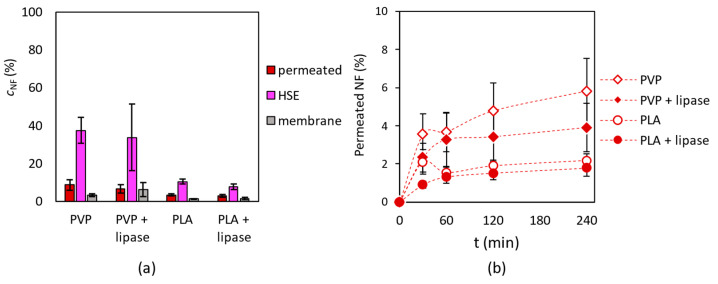
Investigation of nadifloxacin (NF) penetration into human heat-separated epidermis (HSE) applying a topical and transdermal diffusion cell system. (**a**) The concentration of NF in acceptor phase (permeated), in the HSE and in the membrane after 24 h incubation, and (**b**) the amount of permeated NF in a 4 h timeframe at 33 °C.

The HPLC analysis of the acceptor, the donor phases, and the synthetic membrane extracts showed that all nanofibrous masks were able to release nadifloxacin. The highest concentration of the drug was found in the epidermis (HSE) layer. Comparing the enzyme-containing mask to mask without enzymes a slight difference could be observed, while nadifloxacin concentration was a bit smaller in case of enzyme-loaded fibers (Figure 10a). A smaller quantity of nadifloxacin was liberated from PLA-based nanofibers than PVP. The amount of permeated nadifloxacin is continuously increased over the 4 h timeframe with all nanofibrous preparations; PLA fibers showed slower drug release than PVP (Figure 10b). As described in Section 3.4, this is due more to differences in the water solubility of multilayer masks than to differences in their morphology or the strength of nadifloxacin–polymer interactions. During the penetration study the HSE surface was wetted with PBS due to the preparation method, thus PLA may be thought of as a wetted solid layer, whilst the dissolved PVP matrix can be thought of as a dilute gel. Because these systems are distinguished by completely distinct transport mechanisms, the difference between them becomes evident. Solid PLA forms a considerably greater barrier to diffusion, hence the release of nadifloxacin becomes slower [54,55,56]. The difference between enzyme loaded masks and masks with only nadifloxacin could be caused by the formation of a “biofilm” from enzyme molecules, which may lead to an additional diffusion barrier for penetration, although it did not have a remarkable effect. The difference between PVP and PLA can be clearly seen, since PVP dissolved immediately in an aqueous environment, but PLA is insoluble in water, thus more antibiotics remained inside the PLA fibers.

## 4. Conclusions

In this study, a prototype of a novel nanostructured mask was developed using an electrospinning technique. By the sequencing production of three nanofibrous layers with different loadings, such as two types of lipases (lipase from *Candida rugosa* and from *Rizomucor mihei*, CrL and RmL, respectively) and an antibiotic agent nadifloxacin, a combined formula could be achieved. To demonstrate the potential applicability of these masks, water soluble and insoluble polymers, polyvinylpyrrolidone (PVP), and polylactic acid (PLA) nanofibrous layers as candidate structural materials of such a mask were produced and investigated. Results showed that the entrapment was successful, and the entrapped lipases had two orders of magnitude higher specific enzymatic activity than their native form in enzyme-catalyzed hydrolysis of three natural fatty acid (palmitic acid, oleic acid, and linoleic acid) methyl esters. Permeability studies on heat-separated epidermis (HSE) from human abdominal skin confirmed that all nanofibrous mask (simple nadifloxacin loaded or combined mask with lipases and nadifloxacin) could deliver the nadifloxacin to the human epidermis. Our results suggest that the activity of lipases during formulation is strongly influenced by the matrix polymer used and the material quality of the co-formulated drug. The influence on activity is presumably due to the formation of second-order interactions and changes in enzyme conformation due to allosteric modification, but, as mentioned earlier, accurate characterization requires the use of computational chemistry methods. Our previous research has already shown the beneficial effects of hydrogen bonding with cyclodextrin additives for lipases entrapped in polymer nanofibers [42].

Concerning the potential applicability of the novel multi-material nanomask, acne vulgaris which causes an increased sebum production on skin surface, lipase containing formulations could support the drug delivery due to their peeling effect. Due to the enhancing effect of nanofibers on lipase activity and the fine tunable layer production, an electrospinning technique could be a unique tool for advanced skin care.

## Figures and Tables

**Table 1:** Average fiber diameter of different polymeric (PVP or PLA) nanofibers: nanofibers without loading agent, CrL, RmL, or nadifloxacin (NF); loaded nanofibers; and multilayered mask built up from enzyme and antibiotic loaded nanofibrous layers. The diameters of the fibers were determined via software analysis of SEM images. In each case, the average fiber diameters were calculated by measuring 100 independent fiber diameters.

	Formulated Agent				
Polymer Matrix	-	CrL	**RmL**	**NF**	**Multilayered Mask**
	Fiber Diameter (nm)				
PVP	359 ± 68	157 ± 44	233 ± 55	612 ± 233	279 ± 70
PLA	1563 ± 230	1110 ± 378	904 ± 333	945 ± 340	1109 ± 297

**Table 2:** Characterization of PVP and PLA nanofibrous matrices without loading and loaded with lipases (RmL, CrL) and nadifloxacin (NF): the effect of loading components on the Young’s modulus €, contact angle (*CA*) and Surface free energy (*SFE*) of nanofibers.

Polymer Matrix	Loading	*E* (MPa)	*CA*_water_ (°)	*SFE* (mN m^−1^)
PVP	–	92.5 ± 3.8	5.6 ± 0.9	81.2 ± 0.5
	RmL, CrL, NF	13.7 ± 3.2	11.7 ± 1.2	79.9 ± 0.4
PLA	–	22.4 ± 2.3	123.6 ± 2.9	39.7 ± 0.9
	RmL, CrL, NF	10.0 ± 0.2	112.6 ± 2.9	42.3 ± 0.7

**Table 3:** Investigation of the biocatalytic activity (*U*_B_) of lipases (CrL or RmL) formulated in polymeric nanofibers (PVP or PLA). The effect of multilayered nanofiber fabrication and nadifloxacin (NF) loading on *U*_B_ in lipase catalyzed hydrolysis of fatty acid methyl esters (**PA-ME**: methyl palmitate, **LA**-**ME**: methyl linoleate and **OA-ME**: methyl oleate). This table summarizes the specific biocatalytic activity values obtained by gas chromatography of samples taken 2 h after the start of the reactions. Activity values were determined by the following equation: *U*_B_ = *n*_s_ × *c* × (*t* × *m*_B_)^−1^, where *c* is the conversion (%) *n*_S_ is the amount of substrate [µmol], *t* is the time [min], and *m*_B_ is the mass of biocatalyst [g].

Polymer Matrix	Loading	*U*_B_ (U × g^−1^)		
		PA-ME	LA-ME	OA-ME
PVP	RmL	231 ± 59	213 ± 96	290 ± 48
	CrL	209 ± 40	54 ± 7	280 ± 31
	RmL, CrL	192 ± 39	221 ± 9	146 ± 42
	RmL, CrL, NF	28 ± 4	256 ± 6	208 ± 67
PLA	RmL	453 ± 1	375 ± 28	408 ± 40
	CrL	389 ± 39	413 ± 1	418 ± 35
	RmL, CrL	343 ± 40	331 ± 1	350 ± 24
	RmL, CrL, NF	182 ± 49	316 ± 27	345 ± 30

## Data Availability

Data are contained within the article.
